# The effect of a severe psychiatric illness on colorectal cancer treatment and survival: A population-based retrospective cohort study

**DOI:** 10.1371/journal.pone.0235409

**Published:** 2020-07-29

**Authors:** Alyson L. Mahar, Paul Kurdyak, Timothy P. Hanna, Natalie G. Coburn, Patti A. Groome

**Affiliations:** 1 Department of Community Health Sciences, Manitoba Centre for Health Policy University of Manitoba, Winnipeg, Manitoba, Canada; 2 ICES, Toronto, Ontario, Canada; 3 Department of Psychiatry, University of Toronto, Toronto, Ontario, Canada; 4 Division of Cancer Care and Epidemiology, Department of Oncology, Queen’s University, Kingston, Ontario, Canada; 5 Department of Surgery, Division of General Surgery, University of Toronto, Toronto, Ontario, Canada; 6 Division of Cancer Care and Epidemiology, Department of Public Health Sciences, Queen’s University, Kingston, Ontario, Canada; Universita degli Studi dell'Insubria, ITALY

## Abstract

**Objectives:**

To identify inequalities in cancer survival rates for patients with a history of severe psychiatric illness (SPI) compared to those with no history of mental illness and explore differences in the provision of recommended cancer treatment as a potential explanation.

**Design:**

Population-based retrospective cohort study using linked cancer registry and administrative data at ICES.

**Setting:**

The universal healthcare system in Ontario, Canada.

**Participants:**

Colorectal cancer (CRC) patients diagnosed between April 1st, 2007 and December 31st, 2012. SPI history (schizophrenia, schizoaffective disorders, other psychotic disorders, bipolar disorders or major depressive disorders) was determined using hospitalization, emergency department, and psychiatrist visit data and categorized as ‘no history of mental illness, ‘outpatient SPI history’, and ‘inpatient SPI history’.

**Main outcome measures:**

Cancer-specific survival, non-receipt of surgical resection, and non-receipt of adjuvant chemotherapy or radiation.

**Results:**

24,507 CRC patients were included; 482 (2.0%) had an outpatient SPI history and 258 (1.0%) had an inpatient SPI history. Individuals with an SPI history had significantly lower survival rates and were significantly less likely to receive guideline recommended treatment than CRC patients with no history of mental illness. The adjusted HR for cancer-specific death was 1.69 times higher for individuals with an inpatient SPI (95% CI 1.36–2.09) and 1.24 times higher for individuals with an outpatient SPI history (95% CI 1.04–1.48). Stage II and III CRC patients with an inpatient SPI history were 2.15 times less likely (95% CI 1.07–4.33) to receive potentially curative surgical resection and 2.07 times less likely (95% CI 1.72–2.50) to receive adjuvant radiation or chemotherapy. These findings were consistent across multiple sensitivity analyses.

**Conclusions:**

Individuals with an SPI history experience inequalities in colorectal cancer care and survival within a universal healthcare system. Increasing advocacy and the availability of resources to support individuals with an SPI within the cancer system are warranted to reduce the potential for unnecessary harm.

## Introduction

The association between a severe psychiatric illness (SPI) and worse cancer survival has received less attention than other physical diseases, although physical illness is the leading cause of death among people with mental health disorders [1]. Individuals with an SPI likely have a similar cancer burden, yet higher than expected case-fatality rates [2–10]. The majority of evidence supporting this conclusion is based on a comparison of mortality rates in individuals with a psychiatric illness to a non-psychiatric population, rather than in comparing survival within cancer populations or is hampered by low study power and inappropriate control for intermediate variables on the causal pathway. Multiple cancer sites are sometimes grouped together in order to get an adequate sample size, despite the high probability that cancer-related prognosis is likely not uniformly worse across cancer sites [10–16]. Studies also control for many variables that make up the constellation of factors contributing to vulnerability, including socioeconomic status, which may underreport the magnitude of association [12, 13].

Differences in cancer survival may be explained by the non-receipt of guideline-concordant cancer treatment. Although understudied, there is evidence of disparities in cancer care provided to individuals with an SPI across the cancer continuum, from screening to palliative care. Review articles, case reports, opinion pieces and case series make up a significant portion of the literature that are the basis of cancer care recommendations for patients with an SPI [3–5, 17–22]. A small amount of research has been performed to understand if individuals with an SPI are more likely to receive suboptimal oncology care [12, 14, 23–31], or investigated specific barriers to providing cancer care to individuals with a serious mental illness [32]. These studies consistently document the suboptimal cancer treatment of individuals with an SPI and cancer. Few have investigated treatment for a single cancer site within the context of clinical guidelines [23–25, 30], which limits the interpretation and application of the study results to clinical practice.

Clearer evidence around potential cancer care disparities for individuals with an SPI is needed to form the basis of clinical management and health policy reform. Therefore, the objectives of this study were to (1) investigate the effect of an SPI history on colorectal (CRC) survival; and 2) investigate the association between an SPI history and guideline recommended CRC treatment within subsets of eligible patients to identify potential inequalities along the cancer continuum contributing to worse outcomes.

## Materials and methods

### Study design & population

This retrospective cohort study linked administrative health data from the province of Ontario, Canada. Ontario is the most populous province in Canada and residents are eligible for universal health coverage and receive cancer care within the public health system. The study cohort included Ontario residents aged 18 or older who were diagnosed with malignant colorectal cancer between 04/01/2007 and 12/31/2012 (International Classification of Disease, 9th Revision (ICD-9) codes for primary cancer site (153.0–153.4, 153.6–153.9 154.0–154.1) and ICD-O-3 behaviour codes (malignant = 3)). Cancer cases were identified through the Ontario Cancer Registry (OCR), which contains information on 98% of cancer diagnoses in Ontario from 1964 onwards [33, 34]. Individuals who met any of the following criteria were excluded from the cohort: simultaneous colon and rectum tumour presentation; a previous cancer diagnosis; a cancer diagnosis listed on their death certificate only; or less than six months of Ontario Health Insurance Plan (OHIP) coverage prior to diagnosis. Ethics approval was granted by the Queen’s University Health Sciences Research Ethics Board in Kingston, Ontario, Canada. Data were completely de-identified for use in this study. Individual informed consent was waived. There was no public or patient involvement in the study design or execution. The STROBE reporting guideline was followed [35].

### Data sources

These datasets were linked using unique encoded identifiers and analyzed at ICES (formerly the Institute for Clinical Evaluative Sciences). We accessed the following databases: the Canadian Institute for Health Information (CIHI) Discharge Abstract Database (DAD) and the Ontario Mental Health Reporting System (OMHRS), which contain details on all psychiatric hospital admissions in the province; the OHIP database and the ICES Physician Database, which contain physician billing data and physician speciality information; the National Ambulatory Care Reporting System (NACRS), which includes information on emergency department visits; Cancer Care Ontario’s Activity Level Reporting database which includes information on all radiation delivery; and the Registered Persons Database, which includes demographic information and vital status. The Ontario Registrar General database provided information on cause of death.

### Variables

#### Severe psychiatric illness measurement

SPI history was the main exposure variable. We developed an algorithm to identify individuals with SPI in administrative records [36–39] and defined an SPI history as hospitalizations, psychiatry visits, and psychiatric emergency department visits in the six months to five years preceding the cancer diagnosis with a diagnosis of major depression, bipolar disorder, schizophrenia, or other non-organic psychotic illnesses [40] (S1 and S2 Tables). A lag time of 6 months before the start of exposure collection was established to improve the likelihood that the mental illness diagnosis was not related to a new cancer diagnosis. Individuals who were hospitalized for SPI were assigned inpatient SPI status. Individuals who were not hospitalized, but had two or more visits to a psychiatrist or an emergency department with an SPI, were assigned outpatient SPI status. Individuals with inpatient or outpatient SPI history were studied separately to capture an SPI severity gradient [41, 42]. This definition aligns with recommendations for measuring an SPI in the absence of functional status and disability data [43, 44]. Individuals who did not meet the definition of SPI, but showed evidence of another mental illness (e.g., other hospitalizations, physician visits or emergency department visits for mental illnesses not included in our SPI diagnostic list) were excluded from the study (S1 & S2 Tables).

#### Outcome measurement

The primary outcome variables were cancer-specific survival and non-receipt of guideline-recommended surgical resection and adjuvant treatment. Overall survival or death from any cause was also measured. Death clearance data were available to October 31, 2015. Cause of death data were available to December 31, 2012 and capture rates are almost 100% given mandatory reporting [45]. Follow-up time was censored at December 31, 2012 for analyses of cause-specific death and on October 31, 2015 for all-cause death. Cancer-specific survival time was defined as the interval occurring between the date of diagnosis and the date of cancer-specific death. The antecedent cause of death was categorized according to ICD-9 disease level headings and cancer deaths were considered the primary event. Overall survival time was defined as the interval between the date of diagnosis and date of death from any cause.

Non-receipt of guideline recommended treatment was the secondary outcome and measured as two separate dichotomous variables: non-receipt of surgical resection (yes/no) and non-receipt of adjuvant treatment (yes/no). These outcomes were studied in stage and tumour location stratified cohorts, consistent with clinical guidelines [46–49]. Surgical resection was measured using physical billing records and identified using the following codes: S166, S167, S168, S169, S170, S171, S172, S173, S188, S213, S214, S215, S217 in the year following diagnosis. Receipt of adjuvant chemotherapy was measured using physician billing records and defined as the presence of at least one of the following codes within the six months following resection: G381, G345, G281, G339, G359, and G382 [50]. Receipt of adjuvant radiation was defined as the presence of at least one radiation treatment record coded for adjuvant or curative treatment to the pelvis or rectum, in the six months preceding or following surgical resection.

#### Covariates

This work was situated within a causal framework hypothesizing pathways from an SPI to worse cancer outcomes, developed using directed acyclic graph theory [51]. We hypothesized that psychiatric symptoms, treatment, consequences, as well as bias and stigma affected cancer survival and receipt of treatment through multiple pathways (S1 Fig), including reduced access to physical healthcare, medical contraindications, a lack of patient centred care, and institutionalized stigma [52]. Potential covariates were identified through a literature review and the causal framework was used to identify their role (causal pathway, confounder) in the relationship to avoid underestimating the total effect. We considered age at cancer diagnosis, sex, rurality, year of cancer diagnosis, TNM stage at diagnosis (treatment outcomes only), and primary tumour location to be measurable confounders. Major and minor physical comorbidities, social vulnerability factors (e.g., income, education) and TNM stage (survival outcomes) were considered causal pathway variables.

Age, sex, year of diagnosis, stage and tumour location were captured in the OCR. Cancer stage was operationalized as broad categories mapping onto the 6th and 7th editions of the Union for International Cancer Control/American Joint Committee on Cancer (UICC/AJCC) classification system [53, 54]. Tumour location was categorized as colon or rectum using ICD-9 codes. Rurality was estimated using the Rural Index of Ontario (RIO) score, an ordinal measure that reflects relative differences in geographic isolation that may impact health and healthcare [55, 56].

Physical co-morbidities were measured from hospitalization, emergency department, and physician billing data in the six to eighteen months prior to the cancer diagnosis using the 32 John’s Hopkins Aggregate Diagnosis Groups (ADGs) [57]. Six ADGs were classified as ‘Major’ physical ADGs and 22 ADGs were classified as ‘Minor’ physical ADGs based on information on the type, diagnosis, and number of encounters and interventions [57]. Quintiles for the four dimensions of the Ontario Marginalization Index (community residential instability, material deprivation, dependency, and ethnic concentration) were measured from Census data linked to postal code [58, 59] and used as proxy measures for individual level marginalization.

### Statistical analyses

We used Kruskall-Wallis tests to compare skewed continuous data and Chi-square tests for independence to compare categorical variables. We plotted the cumulative incidence of death from cancer-specific and non-cancer specific death, using the two-step approach, which is a function of both the overall survival function and the cause-specific hazard [60]. Differences in the cumulative incidence of cancer-specific death were compared using the non-parametric modified chi-square test [61]. We created Kaplan-Meier curves and used Wilcoxon rank sum tests to test stratified differences in overall survival.

Cause of death was dichotomized as cancer-related/other-cause for the competing risks analysis to examine differences in cancer-specific survival. Individuals were censored at the end of their OHIP eligibility, on the date of non-cancer death, or the end of the follow-up period, whichever came first. The association between an SPI history and risk of cancer-specific death was initially estimated using bivariate and multivariable cause-specific and sub-distribution hazards regression (240). There were negligible differences between the two approaches; therefore, the final relative HR and 95% confidence intervals were computed using the cause-specific approach adjusting for age (continuous), sex, tumour location (colon/rectum), and rurality (0–9, 10–30, 31–45, 46–55, 56–75, 75+, Unknown) (238). The association between SPI history and risk of death from any cause was estimated using Cox-Proportional hazards regression. The proportional hazards assumption was evaluated by plotting the standardized scores of the cumulative Martingale residuals [62].

Logistic regression was used to estimate odds ratios that approximated relative risk and 95% confidence limits (non-receipt surgical resection) and modified Poisson regression with robust error variance was used to estimate relative risks and 95% confidence limits (non-receipt of adjuvant treatment). The analysis for non-receipt of surgical resection was restricted to CRC patients with stage II and III cancer. The analysis for non-receipt of adjuvant treatment was restricted to resected stage III colon and resected II and III rectal cancers. Age (<45, 45–54, 55–64, 65–74, 75–84, 85+), sex, primary tumour location (colon/rectum), stage at diagnosis (II, III), RIO score (0–9, 10–30, 31–45, 46–55, 56–75, 75+, unknown), and year of diagnosis (2007, 2008, 2009, 2010, 2011, 2012) were included as covariates in the adjusted analyses. Age was modeled as a nominal, categorical variable to allow for non-linear associations with each outcome. Model fit was inspected by assessing the Hosmer-Lemeshow goodness of fit statistic. Small statistics and large p-values indicate better model fit. Residual plots were used to visually inspect model diagnostics.

The study had adequate power (>90%) to detect differences of at least 10% in the non-receipt of surgery and overall 5-year survival between the inpatient SPI group and the non-SPI group.

#### Sensitivity analyses

There is the potential for misclassification of disease status when administrative data sources are used [63]. The robustness of the findings was evaluated by re-analyzing the relationship between SPI history, survival and receipt of cancer treatment according to six different definitions that included (1) a two-year timeframe to evaluate SPI status; (2) a minimum of 4 outpatient visits for positive outpatient SPI status; (3) family physician visits as part of the positive outpatient SPI status; (4) including single outpatient visits in the reference group rather than excluding them from the study; (5) including individuals with family physician mental health visits only in the reference group; (6) classify individuals by healthcare use alone rather than including only those with particular diagnoses like schizophrenia (e.g., all individuals with a psychiatric hospitalization in the inpatient group).

## Results

**Fig 1:**
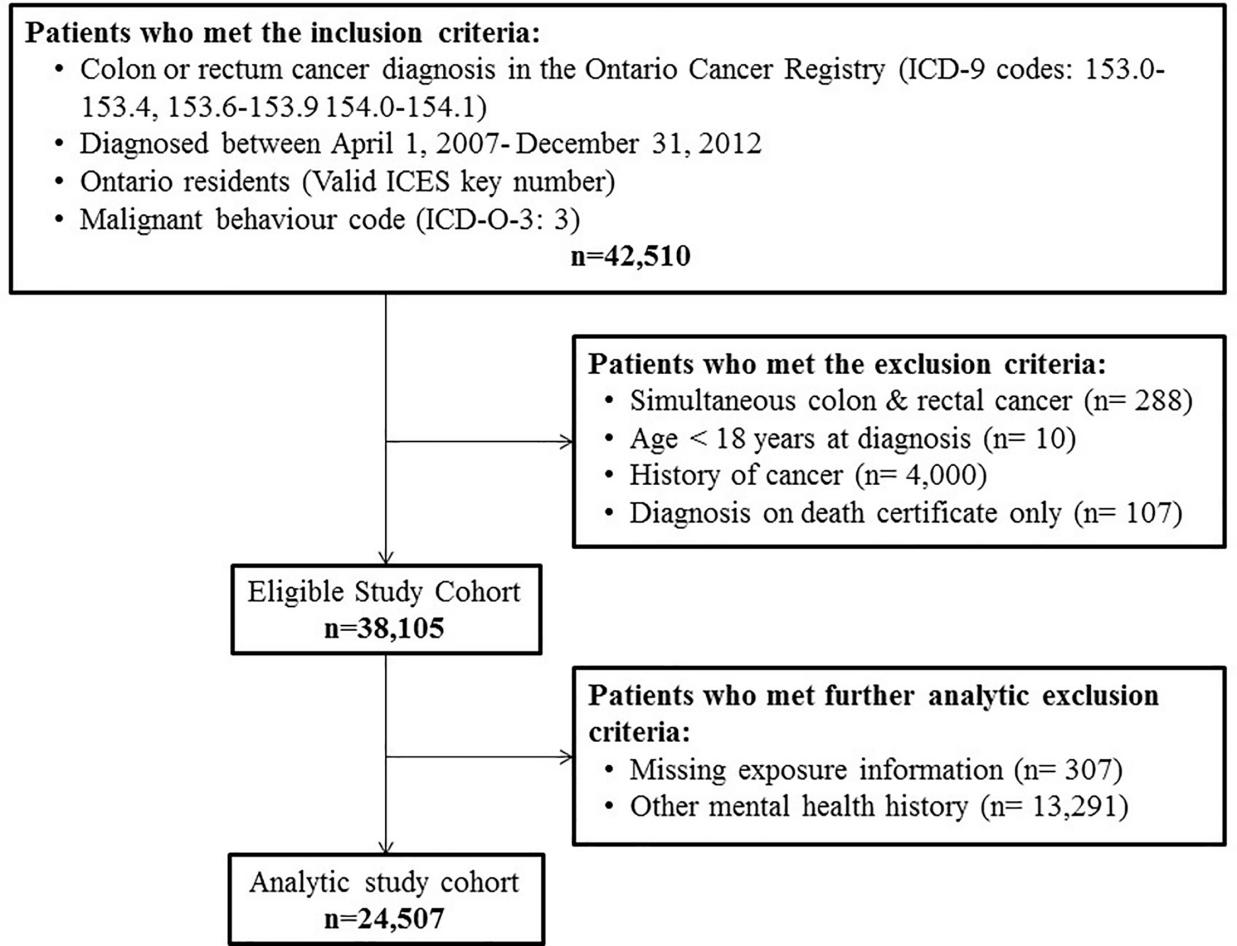
Flowchart of cohort creation process. ICD = International Classification of Disease, O = Oncology.

This study included 24,507 colorectal cancer patients (Fig 1) of whom 482 (2.0%) had an outpatient SPI history and 258 (1.0%) had an inpatient SPI history. Table 1 describes the study cohort. Most patients were diagnosed with an adenocarcinoma located in the proximal colon, rectum, or sigmoid colon. Fewer individuals with an SPI history had cancer stage registered in the data. CRC patients with an SPI history were significantly younger, more likely to be female, had a greater burden of major and minor physical comorbidities, more likely to live in marginalized communities, and less likely to live in rural areas than CRC patients with no history of mental illness.

**Table 1:** Study cohort characteristics (n = 24,507; column %).

	No History of Mental Illness (n = 23,767)	Outpatient SPI History (n = 482)	Inpatient SPI History (n = 258)	p-value^1^
**Histology**				<0.001
Adenocarcinoma	90.6	87.1	83.3	
Non adenocarcinoma	3.9	5.2	4.7	
No histology/NOS	5.4	7.7	12.0	
**Tumour Location**				0.08
Proximal	33.9	35.1	35.3	
Distal	3.4	5.8	3.9	
Sigmoid	20.4	17.4	22.1	
Rectosigmoid	6.9	7.5	4.7	
Rectum	24.4	21.8	21.3	
Other/NOS	11.0	12.4	12.8	
**TNM Stage**				<0.001
0/I	20.0	22.2	18.2	
II	23.2	23.0	20.9	
III	26.1	22.2	23.6	
IV	17.6	16.8	17.4	
Unknown	13.1	15.8	19.8	
**Age at Diagnosis**				<0.001
< 45 years	3.8	5.4	6.2	
45–54 years	10.9	16.2	14.3	
55–64 years	22.0	31.7	27.5	
65–74 years	28.3	22.4	25.6	
75–84 years	25.2	15.6	20.9	
≥ 85 years	9.8	8.7	5.4	
**Female**	41.0	50.8	53.5	<0.001
**Major Physical Comorbidity**				<0.001
0 ADGs	64.3	49.8	45.7	
1 ADGs	26.6	28.0	31.4	
2 ADGs	6.9	15.4	13.6	
3–6 ADGs	2.2	6.8	9.3	
**Minor Physical Comorbidity**				<0.001
0 ADGs	14.5	5.8	6.6	
1 ADGs	17.9	12.4	13.2	
2 ADGs	19.6	15.8	16.7	
3 ADGs	16.9	17.4	12.4	
4 ADGs	12.9	14.9	13.6	
5 ADGs	8.6	12.4	10.9	
≥ 6 ADGs	9.6	21.2	26.7	
**Rurality**				<0.001
0–9 (least rural)	62.9	72.2	65.1	
10–30	18.2	15.6	13.6	
31–45	10.3	8.9	10.1	
46–55	2.9	s	3.1	
56–75	3.0	2.3	3.9	
>75 (most rural)	1.3	0.0	s	
Unknown	1.3	s	s	
Community-Level Deprivation^1^				0.003
1 (least marginalized)	22.8	22.4	18.2	
2	22.8	21.4	17.4	
3	21.4	18.9	18.6	
4	17.9	18.5	22.5	
5 (most marginalized)	13.6	17.2	20.9	

Data available on 24,155 CRC patients; s: cell sizes suppressed according to ICES privacy policy; SPI: severe psychiatric illness; ADG: Johns Hopkin’s Aggregate Diagnosis Group; RIO: Rurality Index of Ontario;

^1^p-values calculated using chi square tests for independence

A total of 10,204 deaths occurred during the study period; 150 (58%) in patients with an inpatient SPI history, 227 (47%) in patients with an outpatient SPI history and 9,872 (42%) in patients with no history of mental illness. Median follow-up time was 4.1 years (IQR 1.9 to 6.8 years) among patients who died or lost OHIP coverage during the study period, and 5.5 years (Interquartile Range 4.1 to 7.0 years) among survivors. Cause of death was available for the 7,142 deaths that occurred before December 31, 2012: 107 in CRC patients with an inpatient SPI history and 157 in patients with an outpatient SPI history. Of those who died, eighty-four percent of patients with no history of mental illness died from their cancer, compared with 80% and 79% of those with an outpatient and inpatient SPI history. The median amount of follow-up time in survivors was similar across levels of SPI history (2.6 years in survivors with no history of a mental illness, 2.6 in those with an outpatient SPI history, and 2.6 years in those with an inpatient history).

**Fig 2:**
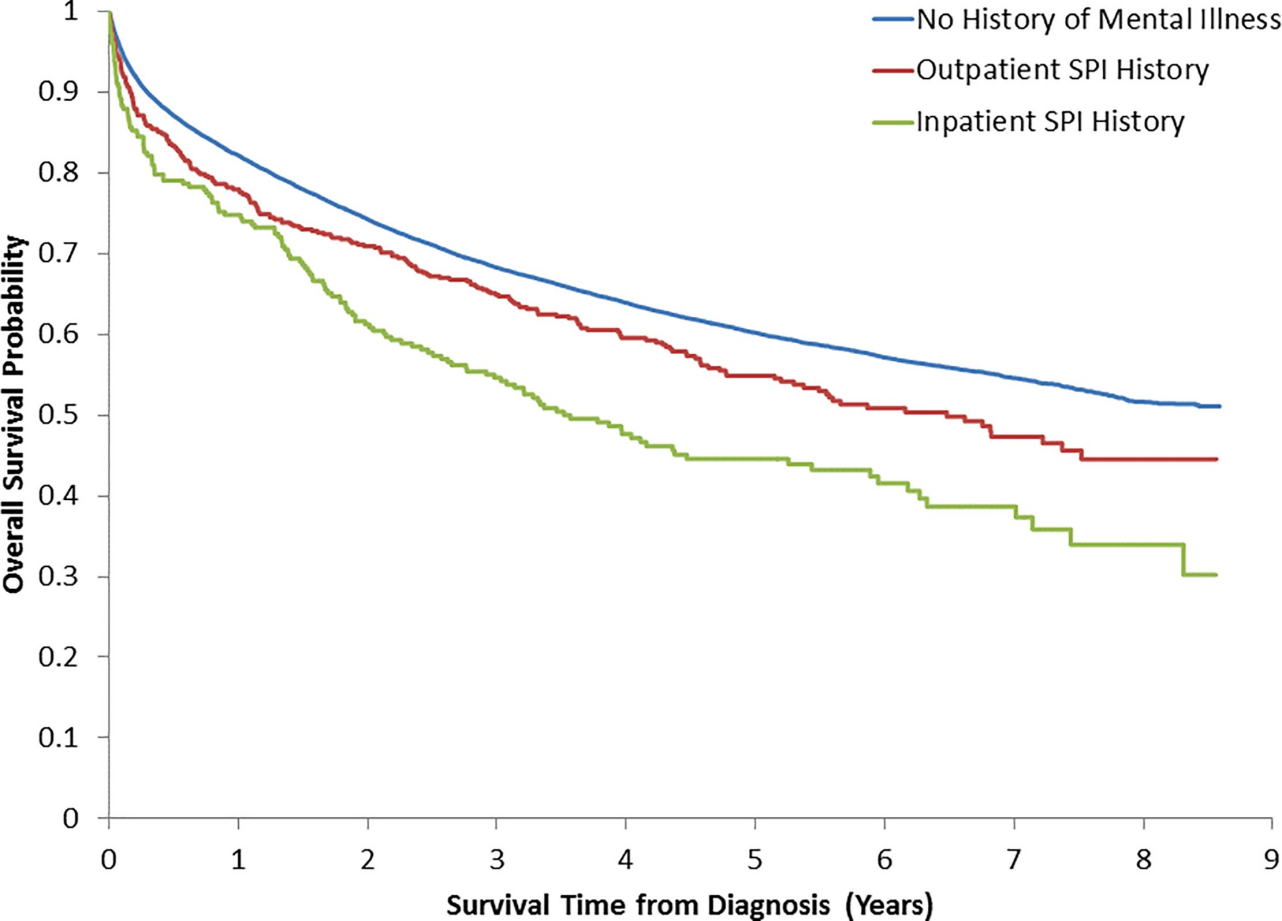
Overall survival across SPI history categories.

**Fig 3:**
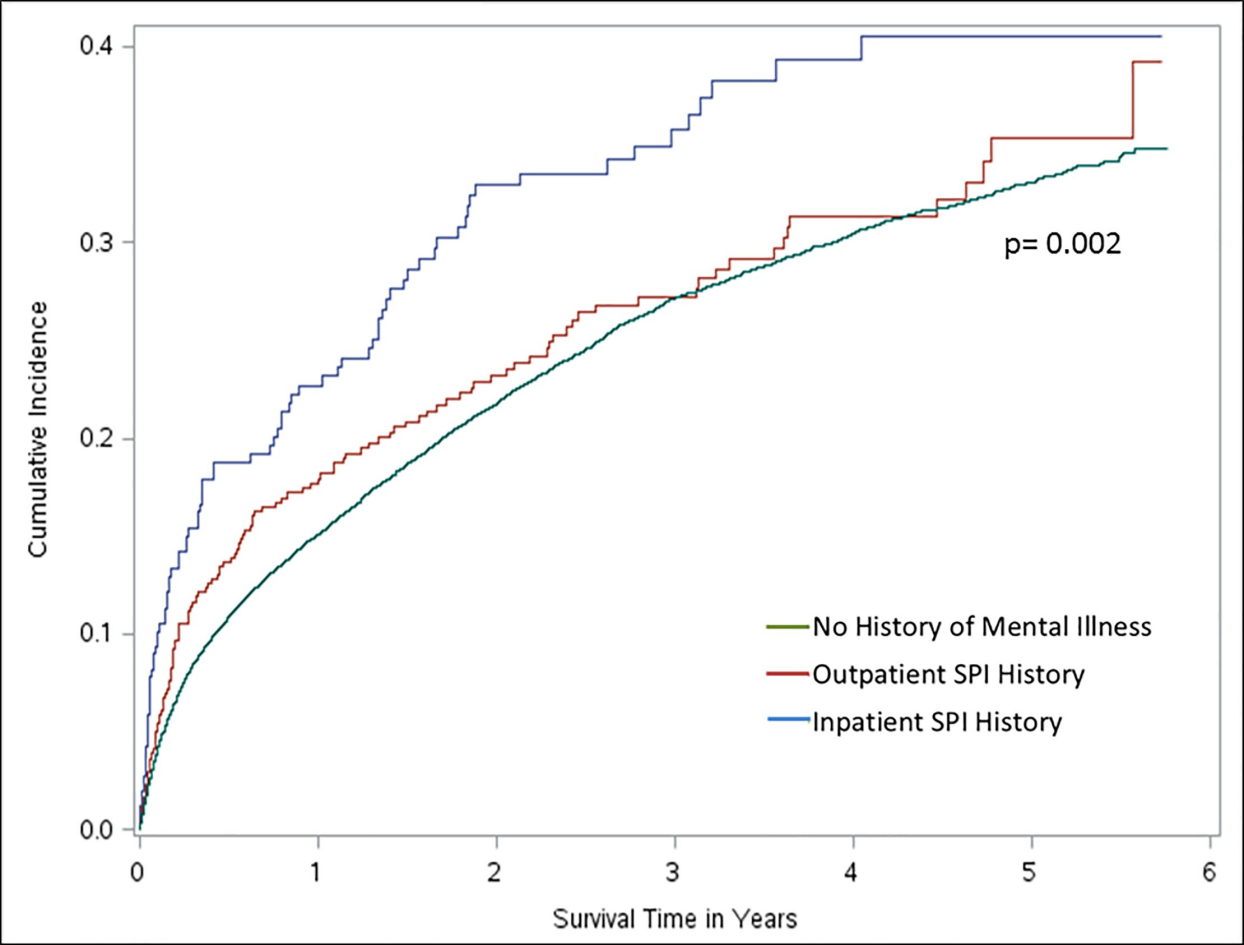
Cumulative incidence of CRC-specific death across SPI history categories.

Five-year overall survival was 45% in CRC patients with an inpatient SPI history, 55% for those with an outpatient SPI history, and 60% in patients with no history of mental illness (Fig 2). Significant differences in survival across SPI history categories were also observed within TNM stage groupings (stage I p = 0.001, stage II p<0.001, stage III p<0.001), with the exception of patients diagnosed with metastatic (stage IV) disease (p = 0.37) (S2 Fig). Cancer patients with no history of mental illness had a significantly lower cancer-specific death incidence than CRC patients with an inpatients SPI (p = 0.002) (Fig 3).

CRC patients with an inpatient SPI history had a 69% higher adjusted risk of cancer-specific death compared to patients with no history of a mental illness (95% CI: 1.36–2.09) (Table 2). CRC patients with an outpatient SPI history had a 24% higher adjusted risk of cancer-specific death compared to patients with no history of a mental illness (95% CI: 1.04–1.48). These results were consistent when death from any cause was evaluated as the outcome (S2 Fig & S3 Table). The proportional hazards assumptions were met for the model including SPI status history and covariates, with the exception of age.

**Table 2:** Association between SPI history and cancer-specific survival using the cause-specific hazards approach (n = 24,507).

	No. (%) cancer deaths	HR (95% CI)	AHR* (95% CI)
**SPI History**			
** **No history of mental illness	5,762 (24.2)	Ref	Ref
** **Outpatient SPI history	125 (25.9)	1.12 (0.93–1.33)	1.24 (1.04–1.48)
** **Inpatient SPI history	85 (32.9)	1.51 (1.22–1.87)	1.69 (1.36–2.09)

HR: hazard ratio; AHR = adjusted hazard ratio; SPI: severe psychiatric illness; CI: confidence interval

*Adjusted for: age, sex, rurality, and tumour location

Non-receipt of surgical resection and non-receipt of adjuvant treatment were investigated in subsets of stage II and III patients. Distributions of demographic characteristics were similar to the overall cohort. Of all eligible stage II and III CRC patients (n = 12,028), 551 (4.7%) of patients with no history of mental illness, 15 (6.9%) of patients with an outpatient SPI history, and nine (7.8%) of patients with an inpatient SPI history did not receive a potentially curative surgical resection. Of eligible, resected stage II and III CRC patients (n = 7,296), 67% with an inpatient SPI history (n = 42) did not receive adjuvant therapy, compared to 44.1% of patients with an outpatient SPI history (n = 56) and 40.3% of patients with no history of a mental illness (n = 2,862).

Individuals with an SPI were significantly less likely to receive either cancer treatment after adjusting for age, sex, rurality of residence, year of diagnosis, tumour location, and stage at diagnosis (Tables 3 & 4). CRC patients with an inpatient SPI history were 2.15 times (95% CI 1.07–4.33) more likely to not receive potentially curative surgery and 2.07 times (95% CI 1.72–2.50) more likely to not receive adjuvant treatment following resection than those with no history of mental illness. CRC patients with an outpatient SPI history were 1.51 times (95% CI 0.88–2.59) more likely to not receive potentially curative surgery and 1.22 times (95% CI 1.00–1.49) more likely to not receive adjuvant treatment following resection than those with no history of a mental illness. Overall, model specification was adequate.

**Table 3:** Association between SPI history and non-receipt of surgical resection (n = 12,028).

	No. (%) no resection	RR (95% CI)	RR* (95% CI)
**SPI History**			
No history of mental illness	551 (4.7)	Ref	Ref
Outpatient SPI history	15 (6.9)	1.49 (0.88–2.54)	1.51 (0.88–2.59)
Inpatient SPI history	9 (7.8)	1.72 (0.87–3.41)	2.15 (1.07–4.33)

SPI: severe psychiatric illness; RR: relative risk; CI: confidence interval.

*Adjusted for age at diagnosis, sex, rurality of residence, year of diagnosis, tumour location, and cancer stage at diagnosis

**Table 4:** Association between SPI history and non-receipt of adjuvant treatment (n = 7,296).

	No. (%) no adjuvant treatment	RR (95% CI)	RR* (95% CI)
**SPI History**			
No history of mental illness	2,862 (40.3)	Ref	Ref
Outpatient SPI history	56 (44.1)	1.09 (0.90–1.33)	1.22 (1.00–1.49)
Inpatient SPI history	42 (66.7)	1.66 (1.39–1.98)	2.07 (1.72–2.50)

SPI: severe psychiatric illness; RR: relative risk; CI: confidence interval.

*Adjusted for age at diagnosis, sex, rurality of residence, year of diagnosis, tumour location, and cancer stage at diagnosis

These results were robust to using alternate administrative data algorithms to measure an SPI history (S4–S6 Tables). The effect estimates for inpatient SPI status were consistent across all sensitivity analyses with a few exceptions. When the time frame for assessing SPI history was limited to two years, the effect estimate for non-receipt of surgical resection decreased (RR 1.01 [95% CI 0.24–4.20]) as did the risk of death (HR 1.67 [95% CI 1.29–2.15]). In addition, when all individuals with psychiatric hospitalizations were included in the inpatient category, regardless of the mental disorder diagnosis, the risk of not receiving adjuvant chemotherapy decreased but remained significant (RR 1.49 [95% CI 1.35–1.64]).

## Discussion

This study used population-based, routinely collected health data to document CRC survival differences in individuals living with schizophrenia, bipolar disorders, major depression and other psychotic illnesses. Across all stages of disease, individuals with an SPI had a significantly higher risk of death than those with no history of mental illness. Some of the survival gap may be explained by a lack of guideline recommended treatment where indicated. Individuals with an SPI had a significantly higher risk of not receiving a surgical resection or adjuvant treatment than those with no history of mental illness. We also identified, for the first time, a gradient in the effect of SPI on cancer outcomes. Individuals experiencing a hospitalization related to their mental illness were the most likely to die from their cancer and the least likely to receive cancer treatment.

This study has a number of strengths and limitations. It is one of very few investigating the relationship between an SPI history and cancer-specific survival and exploring receipt of treatment in a universal healthcare setting. The study benefited from using provincial administrative healthcare data linked to a population-based cancer registry for a single cancer site. This provided the necessary cancer stage and treatment data to produce clinically meaningful and potentially actionable information to oncologists and the mental healthcare team about inequalities in the receipt of care within well-defined, clinical scenarios. The availability of cause of death data also decreased the likelihood that differences in survival may be attributed entirely to differences in baseline physical health or a greater risk of death by suicide. In addition, these data provided a large enough sample size to rigorously address these important questions for such a rare exposure, with adequate study power. The study also separated the SPI effect for individuals with a psychiatric hospitalization, and those with outpatient utilization only and created a comparison group that had no history of mental illness. These exposure measure refinements provided a more detailed and specific estimate of the SPI effect gradient. The current study was restricted to individuals with severe mental illness who are more likely to require psychiatric care in hospital or through a psychiatrist [64, 65]. However, the data sources used to measure an SPI history were physician-centered and did not cover the wide spectrum of community and non-physician services, and so these results may not be generalizable to individuals receiving mental health treatment in the community alone. In addition, the algorithm used to identify SPI history was not validated, which is the gold standard for studies using routinely collected healthcare data to identify positive disease status [63]. However, validation of such a rare exposure poses significant methodological and practical challenges and a similar algorithm [39] has demonstrated good sensitivity and specificity. Multiple sensitivity analyses were performed and we demonstrated our conclusions robust to the chosen definition of SPI.

Our findings of worse cancer survival and lower rates of guideline recommended treatment for CRC patients with an SPI history are consistent with other studies [6, 10–12, 14, 24–26, 30, 66–68]. Variation in the magnitude of effect between our study and others, as well as those finding no association may be the result of a number of methodological differences. Other studies did not use population-based data, dealt with smaller sample sizes, adjusted for factors along the causal pathway such as income, comorbidity, or other vulnerability factors, all of which would lessen the total effect of an SPI. In addition, many did not differentiate based on the severity of mental illness, which would create a lower, averaged effect [6, 12, 14, 25, 26, 30].

The current study provided foundational information on inequalities experienced by individuals with an SPI as well as key gaps in potentially curative treatment, suggesting policymakers should consider how to best integrate psychiatric and oncology care, and improve patient-centeredness of the cancer system. These consistently worse cancer outcomes for a vulnerable population have important implications for all public healthcare systems. SPI patients are less likely to be able to advocate for themselves than patients with no history of mental illness and because of the impact of their psychiatric illness (e.g., low motivation, cognitive impairment, active psychosis), arranging their complex cancer regimens is far more challenging. If the psychiatric disorder interferes with appropriate provision of cancer care, an emphasis on patient-centered care would dictate that the cancer care team identifies ways to provide adequate support throughout cancer treatment. However, limited training, experience, and resources may create difficulties to providing patient-centered care for oncologists and the cancer care team. Consultation with patients, their families, oncologists, oncology nurses, social workers, as well as the key care providers in the mental healthcare system could help inform policymakers on the barriers to ensuring good outcomes for cancer patients with an SPI, or how processes of care can be developed or modified to ensure individuals with an SPI are equally likely to be offered and receive evidence-based cancer care.

Although the body of evidence is sufficient to require system level interventions to improve outcomes for cancer patients with an SPI history, there are a number of areas warranting further investigation to understand how to best implement change. A study investigating the care interface between primary care, psychiatry and non-psychiatry specialties could inform broader interventions targeting changes in healthcare policy and the structure of how healthcare is provided. Evaluations of how existing programs coordinating cancer care, or other types of medical care, for patients with complex needs may be extended to include this vulnerable group, with an understanding that the complexity related to an SPI requires special consideration compared to complexity related to medical comorbidity, are needed. Additional studies investigating other points of care along the cancer continuum, such as palliative care and survivorship care, are needed to complete the picture and better inform clinical management. Future research may focus on the effect of specific mental illnesses, such as schizophrenia and major depressive disorders on cancer outcomes to better understand if particular mental illnesses require customized supports during the cancer experience. Finally, ensuring continuous care of the SPI throughout the cancer diagnosis and treatment periods is critical to continued recovery of the psychiatric illness and also in reducing barriers to cancer care [69]. The results of the current study are likely generalizable to other countries; however, differences in how cancer care is organized and delivered, as well as variations in CRC treatment guidelines or best practices across jurisdictions may result in different, context-specific relationships.

## Supporting information

S1 Fig Causal framework, including summary of causal pathway and confounding variables. (TIF)

S2 Fig Overall survival by severe psychiatric illness history, stratified by stage at diagnosis. a) stage I, b) stage II, c) stage III, d) stage IV.
(DOCX)

S1 Table Diagnosis codes and data sources used to identify relevant healthcare encounters related to a mental illness in the 6 months to five years prior to the colorectal cancer diagnosis. (DOCX)

S2 Table Diagnostic codes used in the exposure assignment algorithm to exclude CRC patients with a non-SPI mental health history from the unexposed group. (DOCX)

S3 Table Association between SPI history and overall survival (n = 24,507). (DOCX)

S4 Table Sensitivity analyses using alternate administrative data algorithms to assign SPI status to study the association between an SPI and receipt of surgery. (DOCX)

S5 Table Sensitivity analyses using alternate administrative data algorithms to assign SPI status to study the association between an SPI and receipt of adjuvant treatment. (DOCX)

S6 Table Sensitivity analyses using alternate administrative data algorithms to assign SPI status to study the association between an SPI and death from any cause. (DOCX)
