# Supplementary material for: The effect of a severe psychiatric illness on colorectal cancer treatment and survival: A population-based retrospective cohort study
Source: PLoS One. 2020 Jul 29;15(7):e0235409. doi: 10.1371/journal.pone.0235409 (PMC7390537; doi:10.1371/journal.pone.0235409)

**S2 Fig. Overall survival by severe psychiatric illness history, stratified by stage at diagnosis a) stage I, b) stage II, c) stage III, d) stage IV**

1.
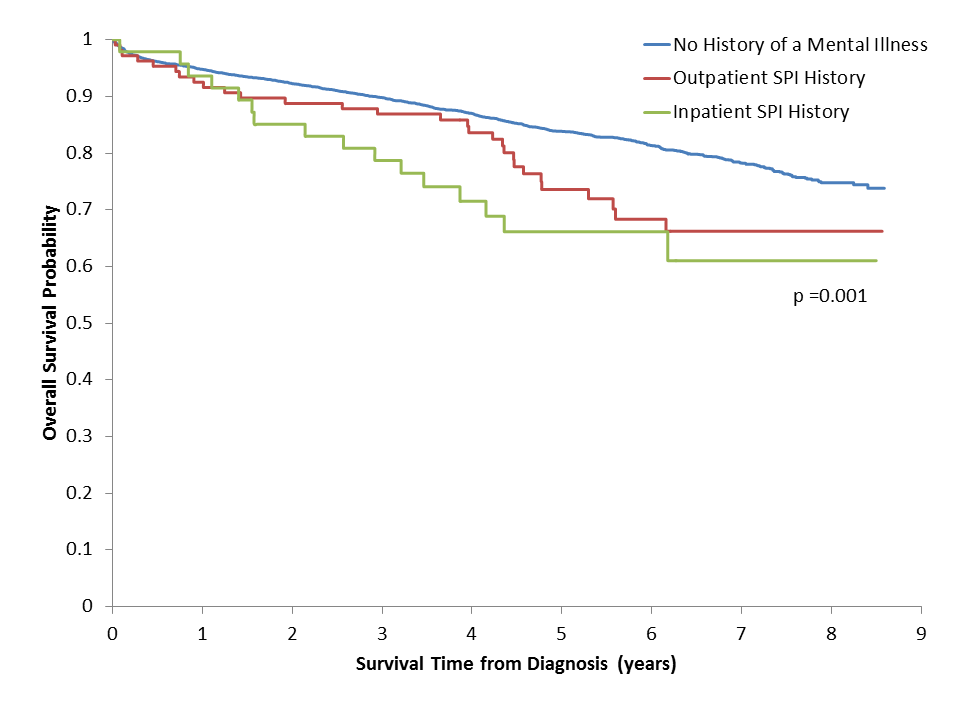

2.
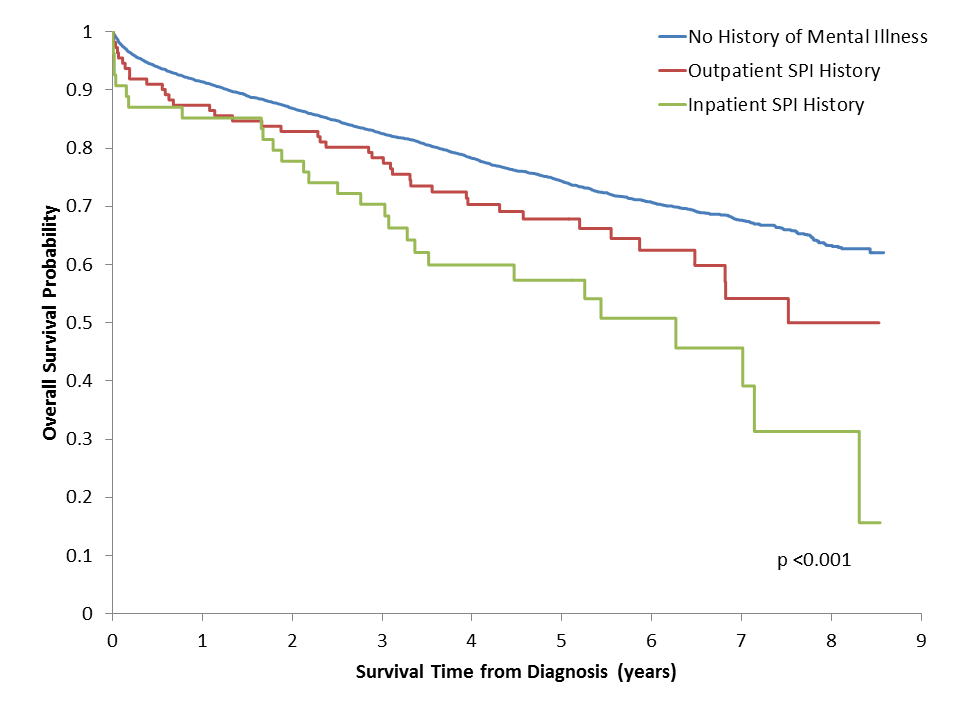

3.
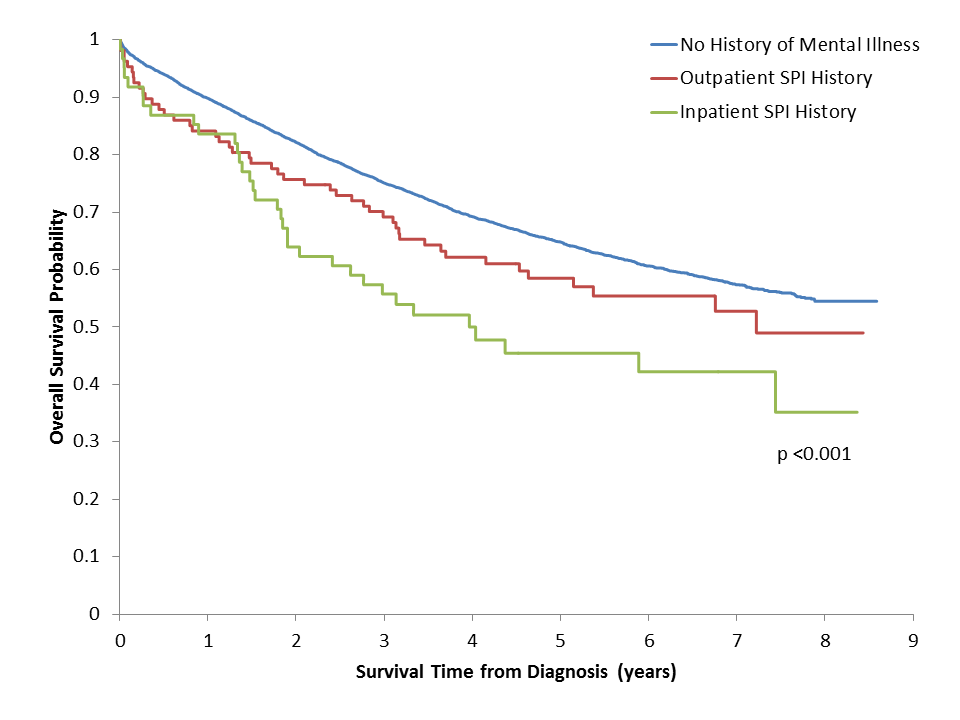

4.
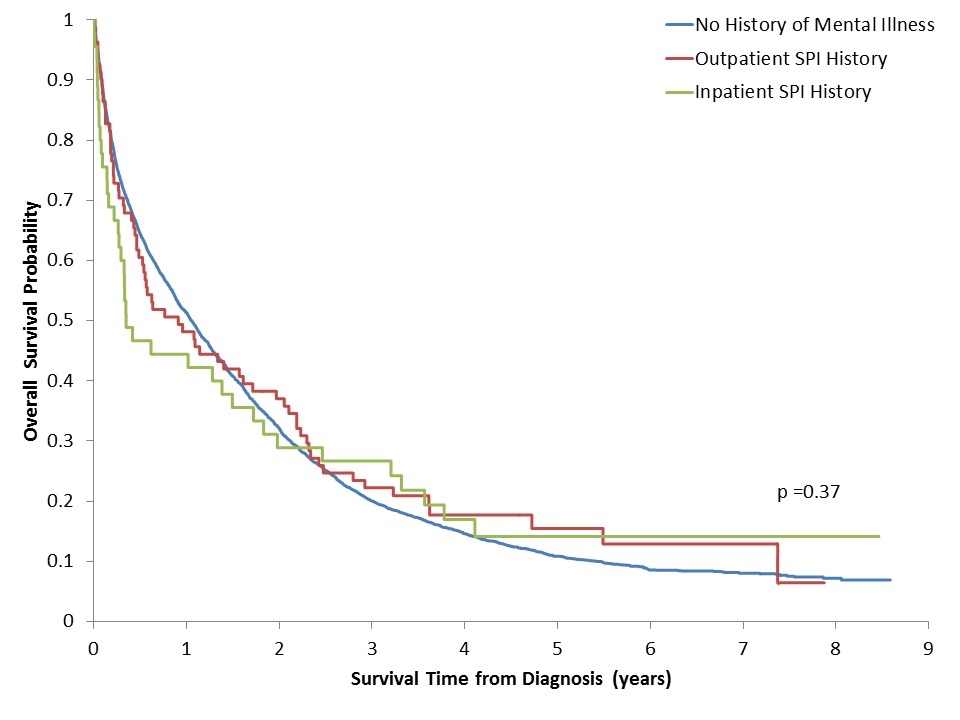

Supplement: S2 Fig — a) stage I, b) stage II, c) stage III, d) stage IV. (DOCX) [file pone.0235409.s002.docx]
