# Supplementary material for: The effect of a severe psychiatric illness on colorectal cancer treatment and survival: A population-based retrospective cohort study
Source: PLoS One. 2020 Jul 29;15(7):e0235409. doi: 10.1371/journal.pone.0235409 (PMC7390537; doi:10.1371/journal.pone.0235409)
Supplement: S1 Table — (DOCX) [file pone.0235409.s003.docx]

**S1 Table. Diagnosis codes and data sources used to identify relevant healthcare encounters related to a mental illness in the 6 months to five years prior to the colorectal cancer diagnosis**

| Database | Diagnostic Code | *Description* |
| --- | --- | --- |
| OHIP^1^ |  |  |
|  | 295 | Schizophrenia |
|  | 296 | Manic Depressive Psychosis, Melancholia Involutional |
|  | 297  298 | Paranoid states  Other psychoses |
|  | 311 | Depressive or Other Non-psychotic Disorder,  Not Classified Elsewhere |
| CIHI-DAD & NACRS^2^ | |  |
|  | F20–F29 | Schizophrenia, schizotypal and delusional disorders |
|  | F20 | Schizophrenia |
|  | F21 | Schizotypal disorder |
|  | F25 | Schizoaffective disorder |
|  | F28 | Other nonorganic psychotic disorders |
|  | F29 | Unspecified nonorganic psychosis |
|  | F30 -F39 | Mood [affective] disorders |
| OMHRS^3^ |  |  |
|  |  | Mood Disorder  Schizophrenia  Other Psychotic Disorder |

^1^Based on ICD-9; ^2^ICD-10 CA; ^3^DSM-IV; OHIP= Ontario Health Insurance Plan; CIHI-DAD= Canadian Institute of Health Information-Discharge Abstract Database; NACRS=National Ambulatory Reporting System; OMHRS=Ontario Mental Health Reporting System
