# Supplementary material for: The effect of a severe psychiatric illness on colorectal cancer treatment and survival: A population-based retrospective cohort study
Source: PLoS One. 2020 Jul 29;15(7):e0235409. doi: 10.1371/journal.pone.0235409 (PMC7390537; doi:10.1371/journal.pone.0235409)
Supplement: S2 Table — (DOCX) [file pone.0235409.s004.docx]

**S2 Table. Diagnostic codes used in the exposure assignment algorithm to exclude CRC patients with a non-SPI mental health history from the unexposed group**

| Database | Diagnostic Code | *Description* |
| --- | --- | --- |
| OHIP^1^ |  |  |
|  | 290  291  292  299  300  301  302  306  307  309  313  314  315  319 | Senile Dementia  Alcoholic Psychosis, Korsakov’s Psychosis  Drug Psychosis  Childhood psychoses (e.g. autism)  Anxiety, neurosis, claustrophobia, depression- reactive, hysteria, neurasthenia, obsessive compulsive disorder, phobias (all types), suicidal tendencies  Personality disorders (e.g., paranoid personality, schizoid personality, obsessive compulsive personality)  Sexual deviations, frigidity  Sexual dysfunction, psychosomatic disturbances  Habit spasms, tics, stuttering, tension headaches, anorexia nervosa, sleep disorders, enuresis, stuttering  Adjustment reaction  Behavior Disorders of Childhood and Adolescence  Hyperactive child, Hyperkinetic Syndrome of Childhood  Specified delays in development (e.g., dyslexia, dyslalia, motor retardation)  Mental deficiency, retardation |
| CIHI-DAD & NACRS^2^ | |  |
|  | F00-F09 | Organic, including symptomatic, mental disorders |
|  | F40-F48 | Neurotic, stress-related and somatoform disorders |
|  | F50-F59 | Behavioural syndromes associated with physiological disturbances and physical factors |
|  | F60-F69 | Disorders of adult personality and behaviour |
|  | F70-F79 | Mental retardation |
|  | F80-F89 | Disorders of psychological development |
|  | F90-F98 | Behavioural and emotional disorders with onset usually occurring in childhood and adolescence |
|  | F99-F99 | Unspecified mental disorder |
| OMHRS^3^ |  | **Variable^4^** |
|  |  | Disorder of Childhood  Delirium, Dementia, Amnestic, Other cognitive Disorder  Mental Disorder due to Medical Condition  Substance-Related Disorder  Anxiety Disorder  Somatoform Disorder  Factitious Disorder  Dissociative Disorder  Sexual, Gender Identity Disorder  Eating Disorder  Sleep Disorder  Impulse-Control Disorder  Adjustment Disorder  Personality Disorder |

^1^Based on ICD-9 (International Statistical Classification of Diseases and Related Health Problems, 9th Revision) ; ^2^ICD-10 CA (Canadian version of the ICD, 10th Revision (ICD-10), created by CIHI); ^3^DSM-IV (Diagnostic and Statistical Manual of Mental Disorders, 4th Edition); ^4^Possible values for each variable included: 0= No, 1= Primary provisional diagnosis, 2= Secondary in importance, 3= Tertiary in importance OHIP= Ontario Health Insurance Plan; CIHI-DAD= Canadian Institute of Health Information-Discharge Abstract Database; NACRS=National Ambulatory Reporting System; OMHRS=Ontario Mental Health Reporting System
