# Supplementary material for: The effect of a severe psychiatric illness on colorectal cancer treatment and survival: A population-based retrospective cohort study
Source: PLoS One. 2020 Jul 29;15(7):e0235409. doi: 10.1371/journal.pone.0235409 (PMC7390537; doi:10.1371/journal.pone.0235409)
Supplement: S3 Table — (DOCX) [file pone.0235409.s005.docx]

**S3 Table. Association between SPI history and overall survival (n=24,507)**

|  | No. (%) deaths | HR  (95% CI) | HR*  (95% CI) |
| --- | --- | --- | --- |
| **SPI History**  No history of mental illness  Outpatient SPI history  Inpatient SPI history | 9,827 (41.3)  227 (47.1)  150 (58.1) | Ref  1.20 (1.05-1.37)  1.63 (1.39-1.92) | Ref  1.40 (1.22-1.59)  1.91 (1.63-2.25) |

HR: hazard ratio; SPI: severe psychiatric illness; CI: confidence interval; *Adjusted for: age, sex, rurality, year of diagnosis, and tumour location
