# Supplementary material for: The effect of a severe psychiatric illness on colorectal cancer treatment and survival: A population-based retrospective cohort study
Source: PLoS One. 2020 Jul 29;15(7):e0235409. doi: 10.1371/journal.pone.0235409 (PMC7390537; doi:10.1371/journal.pone.0235409)
Supplement: S4 Table — (DOCX) [file pone.0235409.s006.docx]

**S4 Table. Sensitivity analyses using alternate administrative data algorithms to assign SPI status to study the association between an SPI and receipt of surgery**

|  | n event^1^  (%) | Adjusted RR  (95% CI) |
| --- | --- | --- |
| Primary Definition  No history of mental illness  Outpatient SPI history  Inpatient SPI history | 551 (4.7)  15 (6.9)  9 (7.8) | Ref  1.61 (0.93-2.77)  2.24 (1.11-4.51) |
| Two Year Timeframe  No history of mental illness  Outpatient SPI history  Inpatient SPI history | 520 (4.8)  7 (5.6)  -- (3.8) | Ref  1.24 (0.57-2.72)  1.01 (0.24-4.20) |
| 4+ Minimum Outpatient Visit Threshold  No history of mental illness  Outpatient SPI history  Inpatient SPI history | 520 (4.8)  9 (6.7)  9 (8.5) | Ref  1.60 (0.80-3.20)  2.48 (1.23-5.03) |
| Include Family Doctor Visits in Outpatient SPI  No history of mental illness  Outpatient SPI history  Inpatient SPI history | 520 (4.8)  24 (4.4)  9 (8.5) | Ref  0.91 (0.59-1.40)  2.46 (1.22-4.98) |
| Ignore Single ED/Psychiatry Visits  No history of mental illness  Outpatient SPI history  Inpatient SPI history | 643 (4.9)  12 (6.0)  9 (8.5) | Ref  1.34 (0.73-2.44)  2.46 (1.22-4.97) |
| Ignore Family Doctor Visit Data (unexposed)  No history of mental illness  Outpatient SPI history  Inpatient SPI history | 791 (4.9)  12 (6.0)  9 (8.5) | Ref  1.34 (0.74-2.48)  2.50 (1.24-5.05) |
| Ignore Diagnosis Codes  No history of mental illness  Outpatient history  Inpatient history | 520 (4.8)  14 (3.0)  63 (15.0) | Ref  0.67 (0.41-1.16)  2.86 (2.25-3.65) |

SPI= severe psychiatric illness;^1^Not receiving surgical resection was considered an event; *Adjusted for: age, sex, rurality, year of diagnosis, tumour location
