# Supplementary material for: The effect of a severe psychiatric illness on colorectal cancer treatment and survival: A population-based retrospective cohort study
Source: PLoS One. 2020 Jul 29;15(7):e0235409. doi: 10.1371/journal.pone.0235409 (PMC7390537; doi:10.1371/journal.pone.0235409)
Supplement: S5 Table — (DOCX) [file pone.0235409.s007.docx]

**S5 Table. Sensitivity analyses using alternate administrative data algorithms to assign SPI status to study the association between an SPI and receipt of adjuvant treatment**

|  | n event^1^ (%) | Adjusted RR  (95% CI) |
| --- | --- | --- |
| Primary Definition  No history of mental illness  Outpatient SPI history  Inpatient SPI history | 2,862 (40.3)  56 (44.1)  42 (66.7) | Ref  1.22 (1.00-1.49)  2.07 (1.72-2.50) |
| Two Year Timeframe  No history of mental illness  Outpatient SPI history  Inpatient SPI history | 2,613 (40.2)  40 (51.3)  19 (70.4) | Ref  1.41 (1.14-1.74)  2.17 (1.72-2.52) |
| 4+ Minimum Outpatient Visit Threshold  No history of mental illness  Outpatient SPI history  Inpatient SPI history | 2,613 (40.2)  32 (42.7)  39 (68.4) | Ref  1.19 (0.92-1.54)  2.08 (1.72-2.52) |
| Include Family Doctor Visits in Outpatient SPI  No history of mental illness  Outpatient SPI history  Inpatient SPI history | 2,613 (40.2)  150 (44.4)  39 (68.4) | Ref  1.18 (1.05-1.33)  2.07 (1.71-2.51) |
| Ignore Single ED/Psychiatry Visits  No history of mental illness  Outpatient SPI history  Inpatient SPI history | 3,214 (40.8)  51 (42.5)  39 (68.4) | Ref  1.22 (0.98-1.50)  2.05 (1.70-2.49) |
| Ignore Family Doctor Visit Data (unexposed)  No history of mental illness  Outpatient SPI history  Inpatient SPI history | 4,033 (41.8)  51 (42.5)  39 (68.4) | Ref  1.20 (0.97-1.48)  2.02 (1.67-2.45) |
| Ignore Diagnosis Codes  No history of mental illness  Outpatient history  Inpatient history | 3,214 (40.8)  133 (42.5)  138 (70.8) | Ref  1.24 (1.09-1.41)  1.49 (1.35-1.64) |

SPI= severe psychiatric illness; ^1^Not receiving adjuvant treatment was considered an event; *Adjusted for: age, sex, rurality, year of diagnosis, tumour location
