# Supplementary material for: The effect of a severe psychiatric illness on colorectal cancer treatment and survival: A population-based retrospective cohort study
Source: PLoS One. 2020 Jul 29;15(7):e0235409. doi: 10.1371/journal.pone.0235409 (PMC7390537; doi:10.1371/journal.pone.0235409)
Supplement: S6 Table — (DOCX) [file pone.0235409.s008.docx]

**S6 Table. Sensitivity analyses using alternate administrative data algorithms to assign SPI status to study the association between an SPI and death from any cause**

|  | n events (%) | Adjusted HR (95% CI) |
| --- | --- | --- |
| **Primary Definition**  No history of mental illness  Outpatient SPI history  Inpatient SPI history | 9,827 (41.3)  227 (47.1)  150 (58.1) | Ref  1.40 (1.22-1.59)  1.91 (1.63-2.25) |
| **Two Year Timeframe**  No history of mental illness  Outpatient SPI history  Inpatient SPI history | 9,827 (41.3)  146 (48.7)  60 (56.1) | Ref  1.50 (1.28-1.77)  1.67 (1.29-2.15) |
| **4+ Minimum Outpatient Visit Threshold**  No history of mental illness  Outpatient SPI history  Inpatient SPI history | 9,827 (41.3)  142 (43.2)  150 (58.1) | Ref  1.21 (1.03-1.43)  1.92 (1.63-2.25) |
| **Include Family Doctor Visits in Outpatient SPI**  No history of mental illness  Outpatient SPI history  Inpatient SPI history | 9,827 (41.3)  623 (47.2)  150 (58.1) | Ref  1.32 (1.22-1.44)  1.92 (1.63-2.25) |
| **Ignore Single ED/Psychiatry Visits**  No history of mental illness  Outpatient SPI history  Inpatient SPI history | 11,946 (41.3)  227 (47.1)  150 (58.1) | Ref  1.41 (1.24-1.61)  1.93 (1.65-2.27) |
| **Ignore Family Doctor Visit Data (unexposed)**  No history of mental illness  Outpatient SPI history  Inpatient SPI history | 14,927 (42.0)  227 (47.1)  150 (58.1) | Ref  1.39 (1.22-1.59)  1.91 (1.63-2.24) |
| **Ignore Diagnosis Codes**  No history of mental illness  Outpatient history  Inpatient history | 9,827 (41.3)  462 (40.0)  785 (71.9) | Ref  1.17 (1.07-1.29)  2.13 (1.98-2.29) |

SPI= severe psychiatric illness; *Adjusted for: age, sex, rurality, tumour location
